# Twenty Years of Unspecified Kidney Donation: Unspecified Donors Looking Back on Their Donation Experiences

**DOI:** 10.3389/ti.2023.10959

**Published:** 2023-02-28

**Authors:** Mathilde C. Pronk, Willij C. Zuidema, Willem Weimar, Jacqueline Van De Wetering, Sohal Y. Ismail, Emma K. Massey

**Affiliations:** Department of Internal Medicine, Erasmus MC Transplant Institute, University Medical Center Rotterdam, Rotterdam, Netherlands

**Keywords:** kidney transplant, anonymity, non-directed altruistic donation, qualitative studies, unspecified kidney donation

## Abstract

The Netherlands was the first European country to implement unspecified kidney donation in 2000. This qualitative study aimed to evaluate the experiences of unspecified kidney donors (UKDs) in our transplant institute to improve the care for this valuable group of donors. We conducted semi-structured interviews with 106 UKDs who donated between 2000–2016 (response rate 84%). Interviews were audio-recorded, transcribed verbatim and independently coded by 2 researchers in NVivo using thematic analysis. The following 14 themes reflecting donor experiences were found: Satisfaction with donation; Support from social network; Interpersonal stress; Complaints about hospital care; Uncertainty about donor approval; Life on hold between approval and actual donation; Donation requires perseverance and commitment; Recovery took longer than expected; Normalization of the donation; Becoming an advocate for living kidney donation; Satisfaction with anonymity; Ongoing curiosity about outcome or recipient; Importance of anonymous communication; Anonymity is not watertight. The data reinforced that unspecified kidney donation is a positive experience for donors and that they were generally satisfied with the procedures. Most important complaints about the procedure concerned the length of the assessment procedure and the lack of acknowledgment for UKDs from both their recipients and health professionals. Suggestions are made to address the needs of UKDs.

## Graphical Abstract

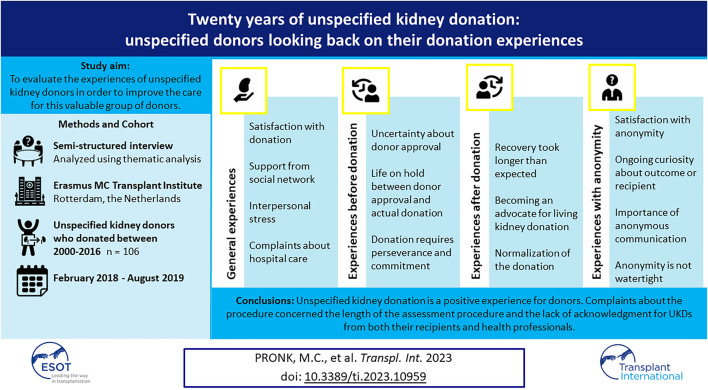

## Introduction

Living donor kidney transplantation is the treatment of choice for patients with kidney failure, because it affords the best patient and graft survival (1). Over the past 2 decades several strategies have been employed to expand the living donor pool, including the introduction of unspecified kidney donation. Unspecified kidney donation refers to living donation whereby an organ is donated by a healthy person to an unknown recipient, i.e., someone they do not know or have ever met. Unspecified kidney donors (UKDs) are also known as non-directed, anonymous, Good Samaritan or altruistic donors (2). An UKD can donate directly to a patient at the top of the waiting list or donate into a kidney-exchange program to trigger a chain of donations (3).

The Netherlands was the first European country to implement unspecified kidney donation in 2000 and since 2005 UKDs have been incorporated into the national kidney exchange program (called domino-paired donation). Currently, the Netherlands and the UK have the highest number of living donor kidney transplants in Europe as well as the highest proportion of UKDs in the living donor pool (4). In the past 5 years, UKDs accounted for 7%–11% of all living donors in the Netherlands (5). The living donor evaluation in the Netherlands follow articles 3–8 of the Dutch Donor Act (6) and guidelines for (anonymous) living kidney donation from the Dutch Transplant Society (available at www.transplantatievereniging.nl/richtlijnen). All living donors in the Netherlands undergo medical and psychosocial screening and compatibility testing. In addition, all UKDs are referred for a mental health assessment by a psychologist or social worker. To ensure privacy of both donor and recipient, the UKD and the recipient remain anonymous before and after the donation. After the transplantation recipients and donors have the possibility to send an anonymous card to each other (*via* the transplant coordinators).

As UKDs currently make an invaluable contribution to the living donor pool, it is important to take good care of this group of donors. Previous literature on the experiences of UKDs worldwide has shown that their donation experience is generally very positive (7-11). Nevertheless, it has also been reported that the donation was experienced as life interrupting or as a source of interpersonal stress (8-12). In addition, some UKDs complained about the intensity and length of the donor assessment procedure and the long waiting time before the actual donation (9-14). Although the aforementioned studies have provided reassuring evidence with regard to the experiences of UKDs, they cannot simply be generalized to the Netherlands, because of different healthcare systems across countries. In addition, there is a need for studies with a longer follow-up time after donation. In our transplant institute we have one of the longest running donation programmes of Europe and as such have one of the largest cohort of UKDs, with a longer follow-up time than reported in previous studies. Therefore, the current study aimed to evaluate the experiences of the cohort of unspecified kidney donors in our transplant institute, which can help to improve the education and care for this valuable group of donors.

## Materials and Methods

### Participants

All UKDs who donated a kidney at the Erasmus Medical Transplant Institute between 2000–2016 were eligible for participation. All donors were above the age of 18 years. Donors were included if they had donated anonymously to the waiting list or through a domino-paired exchange programme. Exclusion criteria were death, therapeutic donors (who underwent nephrectomy for medical reasons) or donation anonymously through the paired exchange program (donors from an incompatible donor-recipient couple). All donors underwent medical and psychological screening, as part of the standardized living donor work-up in our transplant institute.

### Procedures and Measures

All eligible UKDs received a letter from the Erasmus MC Transplant Institute with information on the study. They were called 2 weeks later to assess willingness to participate. If applicable, an interview appointment was made. Between February 2018 and August 2019 donors participated in a semi-structured interview that lasted approximately 45 min. The interview guide (see Supplementary Material) was developed by a multidisciplinary research team consisting of the authors (3 psychologists, 2 nephrologists and 1 former unspecified donor coordinator). Questions covered participants’ experiences with the donor-work up, the hospital admission and recovery period, the reactions from their social environment, and their opinion about the anonymity of the procedure. We also asked whether participants would, in retrospect, make the decision to donate again. Interviews were conducted by the second author (WZ), who was known to all participants through her previous role as unspecified living donation coordinator; however, during the study, she was not involved in the clinical care pathway. Most interviews took place in the out-patient clinic (combined with the yearly check-up). In some cases, data was collected at the donors’ home, depending on participants’ preference, mobility and health. In all settings data was collected individually to ensure privacy. Informed consent forms were signed at the beginning of the interview. Socio-demographic and medical characteristics were obtained from patients records or donor database and checked for accuracy at the beginning of the interview. Ethical approval for the study was obtained from the institutional review board (METC -2017-1180).

### Analysis

Interviews were audio-recorded and transcribed verbatim. Each transcript was anonymized and given a unique study number which was used to identify quotes in this publication. NVivo 12 supported data management and coding. The analysis and reporting of the results conform to the COREQ checklist (see Supplementary Material) (15). Transcripts were coded independently by the first and second author (MP). The first author is a female psychologist (MSc.) with experience in qualitative research. The background of the second author (interviewer) has been described above. An inductive thematic analysis of the transcripts was conducted, in which we followed the six steps described by Braun and Clarke (16). After careful (re)reading of the transcripts, we started with assigning descriptive codes to sections of text that appeared relevant to the research topic. This resulted in an extensive initial code framework. Next, we considered how different codes could be combined into overarching themes or subthemes. Through this process the descriptive codes were redefined and condensed into more meaningful and analytical categories. The data and the code framework were repeatedly scrutinized to ensure that all the significant responses were extracted and allocated to appropriate themes. We carefully reviewed the themes to evaluate if they were coherent and distinct from each other. Each phase of the analysis was extensively discussed by the two coders (MP, WZ) and coding discrepancies were discussed until agreement was reached. When necessary, a third author (EM, psychologist) was consulted. Finally, the themes were described in a narrative form to provide an accurate illustration of each theme. We used words as “many” and “few” to identify the relative frequency of the theme within the study population and to draw attention to (ir)regularities in the data. These words are not meant to convey generalizability beyond the study population.

## Results

### Participants

During the study period 142 UKDs had donated a kidney, either to a patient on the deceased donor waiting list or in an exchange procedure. Eight donors were excluded because they were therapeutic donors and at the time of inclusion 8 donors had died. Cause of death was unrelated to living donation and occurred after a median of 52 months (range 31–164) after donation. Of the 126 remaining eligible donors, 106 gave consent to participate (84%). Reasons for non-participation are outlined in the Supplementary Material. Both positive reasons, such as closure, and negative reasons, such as dissatisfaction, were reported. Socio-demographic and medical characteristics can be found in Table 1.

**TABLE 1:** Socio-demographic and medical characteristics (N = 106).

Socio-demographic characteristics	
Female gender: N (%)	57 (53.8)
Age (years) at donation: median (range)	59 (21–89)
Age (years) at study: median (range)	67 (25–94)
Ethnicity: n (%)	
European	105 (99.1)
Asian	1 (1)
In paid employment: n (%)	56 (52.8)
Highest level of education	
Primary school	5 (4.7)
Secondary/high school	48 (45.3)
Further/higher education	53 (50.0)
Marital status: n (%)	
Married/living together/partnership	61 (51.9)
Single/divorced/widowed	51 (48.1)
Has children: n (%)	65 (61.3)
Has religious affiliation: n (%)	46 (43.4)
**Medical characteristics**	
Time (months) since donation: median (range)	71.50 (23–153)
Registered in deceased donor register: N (%)	92 (86.8)
Registered to donate body to science: N (%)	2 (1.8)

### Themes

**FIGURE 1:**
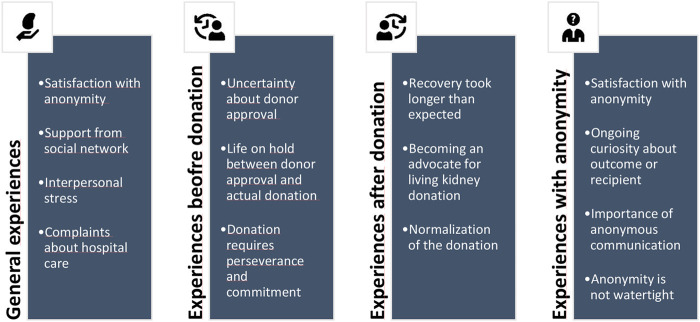
Overview of themes representing the experiences of unspecified kidney donors.

The analysis suggested fourteen themes. We have divided the themes in four categories: general donation experiences, pre-donation experiences, post-donation experiences and experiences with anonymity. Further elucidation of the themes is provided below and an overview of the themes is presented in Figure 1. Tables 2–5 present quotations illustrating the themes.

**TABLE 2:** Illustrative quotations reflecting donors’ general experiences with UKD.

Theme	Quotations^a^
Satisfaction with donation process	“It (the donor work-up) was very well organized. Until the moment of surgery I could say ‘stop!’. I never considered that, but it was all well taken care off.” 110
	“(The donor workup) gave me a feeling of safety; that everything was examined so thoroughly.” 117
	“I found it quite funny and interesting; the whole medical scene. I’ve been operated only once when I was a child, so to experience this whole procedure once is fascinating.” 466
	*“*To this day it (the kidney donation) has been a very good decision and I never regretted it.” 23
	“I find it wonderful that I got to do this [the kidney donation], I felt like I won €100.000, that’s how happy I was about that I could be an altruistic kidney donor.” 291
	“I never regretted it [the kidney donation] and if my kidney would grow back, I’d do it again.” 103
Support from social environment	“After the donation I stayed with my sister for 6 weeks. Everyone wanted to help me, but they also do that when I have a normal flu.” 44
	“Before everyone said ‘gosh, wat are you doing?’ and after the donation they found me very brave and I received a lot of flowers and cards.” 292
	“I like to be by myself, so I did not really get much support and I did not want it, I could manage by myself. I bought groceries beforehand and my son went shopping for me once, but more help I did not get.” 414
	“At work they did not cooperate at all. I work in the healthcare sector, but they were not supportive. (…) It made me very sad.” 337
Interpersonal stress	“My wife first thought I was joking. She did not come to the hospital after I donated. She left the house as a direct consequence of my kidney donation, because I did not give in at all. I told her that even though we were married, my kidney is no part of that.” 136
	“I gave my parents a DVD and leaflets about altruistic donation, but they did not even watch it. I distanced myself from my parents a few weeks before the donation, we had no contact for a while. My parents threatened to sell my horse trailer, they were desperate to stop me from donating.” 486
Negative experiences with hospital care	“No one in the hospital said to me ‘wow, nice that you have done this!‘. They said nothing kind, nothing friendly. One nurse said something nice to me and I suddenly got emotional, but the others only chatted among themselves about their private life. I find that very unprofessional.” 193
	“They were very kind before the donation, but after the donation they were like ‘shut up and stay away’. That’s how I felt it.” 173
	“Only the financial compensation was strange. Why does it have to take so much effort to get my travel costs reimbursed? A living kidney donor saves the health insurance companies almost €50.000. So why do they care about a few euros?” 220

^a^ The numbers at the end of the quotations are identifiers and represent the participant numbers.

**TABLE 3:** Illustrative quotations reflecting donors’ pre-donation experiences.

Theme	Quotations^a^
Uncertainty about donor approval	“It was nice to be turned inside out in preparation for the donation. Other people have to pay for that kind of check-up.” 426
	“At times it was stressful, because you’re afraid they will find something you do not wanna know’” 106
	“I really hoped I would be allowed to donate, because I saw it as a lifesaving act. My major thought was, that I hoped I could do it, that I hoped to pass the tests, despite my mild hypertension.” 298
	“I was only a little nervous about the psychological screening, because the psychologist was hard to read and I did not know what they thought of me.” 397
	“The psychologist asked me a lot of strange questions. He started out with asking why I wanted to donate, if I was sure I did not want it just to receive attention etcetera. I’m not easily taken aback, but the conversation with this psychologist did not feel good.” 193
Life on hold during donor work-up	(Donor who had to wait several months before an operation date): “I mean, you made the decision to donate, you’ve passed the tests, medically and psychologically all is fine. I got the feeling I got stalled, while so many people are waiting for a kidney. Why did it have to take so long?” 424
	“I hoped I would donate in summer, so that I could recover in the garden, but then it got autumn. I felt a little inpatient, that it took longer again. I looked forward to it and then again it did not happen.” 45
	“On the day of admission I felt relieved, it is finally happening, the waiting is over.” 46
Donation requires perseverance and commitment	“The reactions (from the social environment) were mainly positive. Or ‘good that you do that’ and ‘I’d never do it.’. My sister was vehemently against it, because ‘you have two kidneys not without reason, what is something happens with the other kidney?’. But it did not make me doubt my decision or whatsoever, no.” 111
	“People said: ‘Girl, at your age? You’re crazy!’. My kids had lost their dad already, how would it go with their mum? I can imagine that.” 322
	“I constantly had to defend myself, when I told others about my plans to donate. That was hard for me. Not only in my social network, but also in the hospital. When I got my blood drawn and we started chatting, the nurse said: ‘wow, why do you do this? For a stranger?’ It felt like I had to go in defense-mode.” 124
	“I encountered resistance from people. Some people even got angry, so I stopped talking about it. I feel like more education about living donation is needed.” 262
	“Before the donation I had not told many people, because I knew that my sister would not agree. So I thought, I’m not gonna talk about it, because people will only make a fuss about it.” 369
	“I never doubted my decision. I always felt like, this is something personal. I want this and what others think of it, is their opinion. This is my choice and I do not care what others think.” 486

^a^ The numbers at the end of the quotations are identifiers and represent the participant numbers.

**TABLE 4:** Illustrative quotations reflecting donors’ post-donation experiences.

Theme	Quotations^a^
Recovery took longer than expected	“I got problems with the wound and I had to take it slow for a few months. At that point I pitied myself: I donated my kidney, I do not deserve this.” 103
	“I only started to work again after 3 months. I had a lot of pain in my stomach, probably due to scar tissue, and I kept on getting bladder infections, but I recovered. I see other donors who could cycle again after one or 2 weeks, but not me.” 193
Becoming an advocate for living kidney donation	“The reason that I share my donor experience with others is because I want to draw people’s attention to the possibility of becoming a living donor.” 256
	“I’m an active member of my church and together with the pastor I have organized two services about kidney donation. (question for the interviewer): Do you know anything else I could do or participate in?” 102
	(Donor who got featured on several TV channels and in newspapers): “In retrospect I’d have done it differently. I’d have never cooperated with TV, an anonymous newspaper article would have been enough, because I’d have control over that. Looking back on my feelings about the media, it was a media storm. On the other side it has triggered a lot, but I would not work with too many media channels again.” 23
	“I shared a story about my kidney donation of Facebook, to spread the word about living kidney donation.” 339
Normalization of the donation	“I’m surprised that to me it (the unspecified kidney donation) is the most natural thing in the world and others find it extraordinary. I do not understand that people do not do it, to me it is so very logical to donate. I’d prefer to donate another kidney, so to speak.” 102
	“I do not see it like wow I’m such a good person, because I donated my kidney. I do not think about it anymore. I once donated that thing, it’s finished. It’s history.” 268
	“It was a piece of cake. The surgery was on Friday, I was discharged on Monday. I did not notice anything from the surgery or the scar.” 424
	“Being down to earth, I’d say: it’s not that special. Just do it, it’s no effort and you derive a lot of satisfaction in return.” 449

^a^ The numbers at the end of the quotations are identifiers and represent the participant numbers.

**TABLE 5:** Illustrative quotations reflecting donors’ experiences with anonymity.

Theme	Quotations^a^
Satisfaction with anonymous procedure	“I was happy with the anonymity, because the recipient does not have to bother about giving something in return. I rather give than receive presents.” 224
	“I see only advantages of anonymity. If I’d hear the kidney had been rejected by the recipient, I’d be so sad for that person, I’d very upset to hear that.” 226
	“I absolutely do not want to know my recipient. If I’d know, I’d watch that person. If he lives good, eats healthy, and I do not have the right to interfere with another person like that. But if I knew, I might do that.” 372
	“To me anonymous kidney donation is the purest form of donation. No personal interests played a role in my donation. Anonymity has been the force behind my donation.” 23
Ongoing curiosity	“I’d only like to know if the kidney still functions. I do not need to know an age or anything about the recipient, because then the anonymity would be gone.” 23
	“Honestly, I’d like to know who got my kidney. I’d like to know if he/she is doing fine, and if the recipient would want that, I’d like to meet him/her. I regularly think about my recipient and it’s a pity that I do not know anything about the outcome of the donation” 45
	“It’s a pity that you do not get to know if the donation has been successful, because if you do not hear anything, for what did I do it? It’s a shame that I do not know if my goal [helping someone] has been achieved.” 217
Importance of receiving anonymous communication	“I’ve always found it a shame that I did not hear anything. How on earth it is possible that someone receives a kidney and does not even send a postcard or a soap bar or just something, a gesture, I do not understand it.” 268
	“It has struck my mind that I’d have written a card if I’d been the recipient. I’d have been so happy and I’d have wanted to express that. I just find it a little strange. Was receiving my kidney so ordinary for them? Did the transplantation not go well?” 339
	“I received a letter from my recipient. It was very touching and beautiful. I read the letter to my friends and family while celebrating new years eve.” 424
	“It received a card twice. I know it was a young chap and he had been on holiday for the first time in his life, because he was off dialysis. Receiving a postcard from Tenerife was just great!” 369
Anonymity is not watertight	“I was at a donor-day from the patient association and another donor mentioned the date of his donation and I said ‘me too!’. I told him that I received a letter, and then he said ‘it was me writing that letter’ (the other donor participated in a domino-paired donation together with his wife who needed a kidney). I was shocked and touched. His wife was not ready for knowing about me, so we had no contact after that. But I now know that I donated to a mother of four children.” 247
	“I had to wait in a doctor’s room and there were 4 cups on the table. I saw two identical surnames, one foreign surname and my own surname. I just knew that two names belonged to an exchange pair. When I came back for a check-up, that exchange couple was in the waiting room as well for the same check-up!” 424
	“I checked my electronic patient record and at the ‘relatives section’ I suddenly saw a name I did not know. It was the name of my recipient. I could not restrain my curiosity and googled her name. I found everything: how old she was, where she lived and how she was doing at the moment. It is just a coincidence. I mean, I only had the name and decided to google the name. So I have a part in it as well.” 403

^a^ The numbers at the end of the quotations are identifiers and represent the participant numbers.

### General Donation Experiences

#### Satisfaction With Donation

For most donors, the donation was a positive experience. Some called it “interesting” or “amazing.” Donors were generally satisfied with the hospital care and did not experience the donation process as stressful, although they mentioned feeling somewhat nervous before surgery. For some it was reassuring to know that they could withdraw from the process at any time. Most donors experienced a smooth recovery and 98% would make the decision to donate again. The two donors who regretted their decision to donate were dissatisfied about the hospital care they received and criticised the lack of empathy from the hospital staff and/or the reimbursement procedure for donation related expenses.

#### Support From Social Network

The majority of donors felt supported by their relatives and friends during the donation process, despite the resistance they also encountered. During the recovery period participants received both psychological support, (e.g., post-cards), and practical support (e.g., people bringing groceries). Some donors said that they did not need extra support during the recovery, while others would have wanted more support than they received. A few donors reported that contact with other donors *via* patient societies was helpful to them. Only a minority of participants reported on the reaction and support of their employer. Most of them felt supported by their employer, but a few employers did not agree with the decision to donate or donors felt they had to return to work too soon after donation.

#### Interpersonal Stress

A small group of donors mentioned that their choice to become an UKD impacted their relationships, because some loved ones strongly opposed their decision to donate and could not understand it. A few donors said they broke up with their partner, because he or she could not accept the donors’ decision. Several others mentioned conflicts in the parent-child relationship, which led to a temporary loss of contact or a continued estrangement.

#### Complaints About Hospital Care

Despite of the general high satisfaction with the donation process, many participants also had some complaints about their hospital experience. Concerning the donor work-up, some participants mentioned that attending all the appointments caused them inconvenience (some patients had many appointments on 1 day, others had to come to the hospital on multiple days). Concerning the admission for the nephrectomy and the hospital stay, some complained about a level of hygiene, the quality of the food or fellow patients in their shared hospital room. Some participants complained about the responsiveness of and communication with the hospital staff. They felt that their needs (physical or emotional) were unseen or missed genuine interest or empathy from the hospital staff with regard to their donation to a stranger. Some donors felt that they were discharged too soon. A few donors felt frustrated about donation related expenses, such as travel costs, and were dissatisfied about the financial compensation they received. Also, a few donors complained that their family doctor (GP) did not seem to be aware of them only having one kidney.

### Pre-Donation Experiences

#### Uncertainty About Donor Approval

Concerning the donor work-up, donors reported to be happy with the medical check-up. It felt good to be examined so thoroughly. Nonetheless they were nervous about receiving the final test results, because they feared that a reason (medical or psychological) would be found that would prevent them from becoming a UKD. A small group of donors found the psychosocial evaluation strange or intrusive, because of the kind of questions that were being asked (e.g., why they wanted to donate, or if they expected to be praised for their donation by others).

#### Life on Hold Between Donor Approval and Actual Donation

After being approved, donors felt excitement about the upcoming donation, but they still had to wait before a final donation date was set. Some donors reported that the period between the donor work-up and the actual donation was long, which caused problems with the scheduling of work or holidays. A few were anxious something would happen to them in this period of waiting, for example getting ill. Donors were relieved when the donation finally took place.

#### Donation Requires Perseverance and Commitment

When informing others about their decision to become an UKD, donors received both positive and negative responses. People admired them for their remarkable choice to donate, but would not do it themselves. Some loved ones said donating a kidney fits the personality of the donor and, in some cases, friends or family got inspired to become an UKD themselves. For all participants, however, the choice to donate was also met with some resistance and/or concern. Other people found it a risky, or even selfish decision (what if a loved one would need a kidney) and donors were regularly called “crazy” for wanting to become an UKD. Participants reported that they had to constantly justify their decision to donate. To avoid negative reactions, or to protect their loved ones against worrying, many donors waited long to share their decision to donate with others or informed very few people. This sometimes made the donation a lonely process. Despite the negative reactions they received, almost all participants reported that they never doubted their decision.

### Post-Donation Experiences

#### Recovery Took Longer than Expected

Even though the majority of donors, in retrospect, reported that their recovery was smooth and as they expected it to be, for some donors the recovery took longer or was more stressful than expected. Some developed complications, of which wound infections were the most common, or suffered from ongoing pain or exhaustion. A few donors were very unhappy with their scar and underwent scar revision surgery at their own expense.

#### Normalization of the Donation

Many donors mentioned that others perceived their donation as a remarkable act, while for them it was a natural thing to do. They reported that they do not feel special for being an UKD and do not regularly think about the donation anymore. Some do not talk about it anymore, because they do not want or need to be praised for their donation. In retrospect, some donors feel like the donation was not a big deal and that the donation was no effort for them.

#### Becoming an Advocate for Living Kidney Donation

Some participants actively shared their story to create awareness about unspecified kidney donation. When they get the chance, they tell colleagues or other people about the donation. A few others shared their donation experience on social media or participated in educational activities organised by patient foundations. Some donors were asked to share their story on national TV or in a newspaper.

### Experiences With Anonymity

#### Satisfaction With Anonymity

In general, participants were happy with the anonymity of their donation and they understood the advantages of anonymity. They believed that anonymity protected them against an unequal relationship with the recipient or a continued sense of obligation from the recipient to the donor (and *vice versa*). Some donors reported that they did not want gratitude. Donors also said that not knowing the recipient protected them against disappointment if the transplant failed or when the recipient would turn out to be different than they imagined. Finally, participants believed that anonymity ensures an unconditional gift and a fair allocation of organs, based on medical considerations rather than on prejudices. A small group of donors did not agree with anonymity and criticized the secrecy around the recipient, especially after the introduction of the General Data Protection Regulation (a regulation issued by the EU in 2016 to harmonize data privacy laws across Europe).

#### Ongoing Curiosity About Outcome or Recipient

Even though the majority of participants were happy with anonymity, many of them also experienced a level of ongoing curiosity. This curiosity mainly concerned the outcome of the transplantation (does the kidney still function? How is my recipient doing?), but some donors were curious to know (more details about) their recipient. A few would really like to meet their recipient and one donor did actively try to find her recipient. Some participants (repeatedly) called the hospital to inquire about the status of their kidney. During the interview some participants again tried to obtain more information about “their” kidney or about the recipient.

#### Importance of Anonymous Communication

Some donors were informed by their medical doctor that the transplantation had been successful. Knowing that the recipient was doing well meant a lot for these donors and made them happy. Some donors were told some details about their recipient, such as age and/or gender, which was valuable information for them. Only a minority of donors received one (or more) anonymous card(s) from their recipient. They reported that it was satisfying to hear something about the impact of the kidney donation on the recipient’s life. A few donors mentioned that they received a small gift from the recipient, such as a fruit basket or a small amount of money in cash or on a gift card (the highest reported amount was €35). One person received a horseshoe charm, but returned this to the recipient, because he had ambivalent feelings about this gift. Most donors who did not receive an anonymous card from the recipient were not bothered by that, but some would have appreciated a (thank you) letter and were disappointed about not getting one. Some of them were upset, because in their opinion it is the least a recipient could have done to express his or her gratitude through such a card/letter.

#### Anonymity is not Watertight

A few donors had found out who their recipient is, in most cases due to carelessness of health organizations, such as the hospital or health insurance companies. One donor saw the name and address of her recipient in her electronic patient records and another donor was left alone in a doctor’s room with four cups with names on it and could easily figure out who her recipient was. A few others found out the name of their recipients, because they got their travel expenses refunded by the health insurance of the recipient and the insurance companies were not aware of the anonymous nature of the donation. In some cases, donors could make an educated guess about who their recipient was, based on what they saw on the ward or heard from fellow patients (e.g., when sharing a room with the exchange donor in a domino-paired donation).

## Discussion

This large qualitative study summarized the experiences of UKDs in the Netherlands up to on average 7 years after donation and highlighted valuable implications for education and guidance of UKDs throughout the donation process. The donation was predominantly a positive experience for participants and 98% of donors would, if possible, make the same decision again to donate. Most donors were satisfied with the living donor evaluation, with the hospital care they received in the pre-donation and follow-up period, and experienced a smooth recovery and no unexpected or lasting consequences of their donation. These findings are in line with previous research on the experiences of UKDs in the Netherlands and in other countries (7-11). We also found that all participants, to a greater or lesser extent, faced resistance to their choice to become an UKD from friends, relatives or employers, because of a lack of understanding. Similar struggles have been reported by UKDs in other countries, such as the UK (11, 12), Sweden (10), the US (16), and New-Zealand (9). Like in these previous studies, we found that participants responded to the abovementioned struggles by determination and commitment to their decision. A detailed description of the motivation of these donors and the, overwhelmingly positive, impact of the donation on the lives and mental health of this cohort of UKDs can be found elsewhere (17).

Even though we conclude that the donation was generally a very positive experience for the UKDs in our centre, participants also revealed some negative experiences that call for adjustments and improvement of certain aspects of the donation process. Some participants criticized certain procedural aspects, such as the lengthy assessment procedure or the complicated procedures to get a refund for donation-related expenses. Similar complaints are reported by UKDs in other countries (9, 10), but also apply to living related kidney donors as well. It is important to highlight, that participants all donated before 2016 and subsidy regulations in the Netherlands have improved since then. Currently, all living kidney donors are entitled to compensation for donation related expenses (including parking costs, costs for additional medical or homecare, flowers for helpers, travel and accommodation costs for one caregiver) and to partial compensation for loss of income(18). Concerning the length of the living donor assessment procedure, one could try to optimize and shorten the assessment process (although the matching process will always take time, especially when UKDs are included in a kidney exchange program). For example, Northern-Ireland has implemented a one-day assessment process, which resulted in an increase of the living kidney donation rate and in an enhanced overall donor experience (19). Finally, some donors found the psychosocial evaluation disturbing, because they had the impression that their motivation was being questioned, which has also been reported by UKDs in the United Kingdom (13). To prevent these negative experiences, the goal of the psychosocial evaluation should always be explained to the donors. In accordance with the ELPAT living organ donor Psychosocial Assessment Tool (EPAT) (20), currently used in our transplant institute, we stress the importance of emphasizing that the psychosocial assessment is not a test, but an evaluation of how best to prepare for the donation and care for the individual.

On a psycho-social level we found that some donors experienced a lack of social support, an increased tension in relationships during the donation process (e.g., a break-up or estrangements) or a lack of acknowledgement for their donation (from the recipient or from the hospital staff). These are important findings that ask for improvements in the care for these donors, because they might lead to unfavourable psychological outcomes (21, 22). Firstly, assessing the social resources of UKDs should be part of the psychosocial screening for UKDs to identify concerns about a lack of social support or conflicts caused by donation (20, 23). In the EPAT-tool (20) the absence of social support is seen as a red flag for donation and as a signal that education on the impact after donation or additional support from the transplant team is needed. In addition, we recommend to include the social network in the education for and guidance of UKDs throughout the donation process as much as possible (i.e. by actively inviting friends and relatives to accompany the donor to hospital appointments). Although our UKDs were generally happy with the anonymity of their donation, many experienced a level of ongoing curiosity towards the outcome of the transplantation, and, to a lesser extent, towards the identity of the recipient. This curiosity is common among UKDs worldwide (7, 9, 24, 25). While anonymous communication between donor and recipient is allowed in the Netherlands, only a minority of the included donors had received an anonymous card from their recipient. This gesture meant a lot to these donors, because they realized the impact of their act and felt a connection to the recipient (without rescinding anonymity). Some donors who did not receive a card experienced negative feelings about this. Previous studies have also described the importance of receiving acknowledgement from the recipient (9, 10, 12). Although there can be many reasons for recipients not to reach out (ranging from just forgetting about the possibility to a failed transplant), efforts should be made to make recipients of an anonymous living donor kidney more aware of the meaning of anonymous correspondence for their donor. Recipients should be informed about the possibility of sending an anonymous card at least once before the transplantation and once after the transplantation. Finally yet importantly, some participants were disappointed in the attitude of the hospital staff towards UKDs and missed empathy and understanding for their special kind of donation. As also described by Zuchowski and colleagues, who studied UKDs in the UK, the extensive work-up process was in sharp contrast with the treatment donors received after surgery. Although most donors did not want special treatment, they did look for some kind of recognition from the hospital staff (11). ‘It’s like delivering a package, after that you’re just dismissed’, one of our donors said. The disappointment about the lack of this recognition caused some donors to have lasting negative feelings about the donation (17). It should be noted that this feeling of “abandonment” after the donation has also been described by directed living kidney donors (26-29) and has been associated with lower satisfaction rates and a negative influence on quality of life (26). We agree with others in the field that healthcare professionals should “give explicit attention to living kidney donors after the donation”(27-29). Transplant centres should consider how they wish to acknowledge the contribution of anonymous donors, not only through something tangible (such as a card) but also through the attitude of staff. We believe that small actions such as a kind word or a compliment to a living donor will enrich the donor experience, of UKDs in particular. It therefore is important that transplant professionals are educated about the motivations and expectations of UKDs to increase understanding and empathy for this group that makes a major contribution to our public health. The themes found in this study can contribute to the content of such education.

### Limitation of the Present Study and Future Directions

Firstly, a limitation of the study is the retrospective design, whereby findings may be subject to memory lapses or recall bias. Moreover, there is a wide variation in time since donation which we did not take into consideration in the analysis. On the other hand, we believe that we captured the most important experiences of our donors that remained active even years after the donation. It should, however, be kept in mind that participants donated over a long time-frame, in which policies and approaches toward UKDs have changed. Nevertheless we believe that the majority of experiences still apply and should be used as indicators to improve care for these donors. Secondly, the fact that the interviewer was known to the participants, based on her previous role as unspecified donor coordinator, could have introduced bias, for example in an attempt to avoid disappointment or embarrassment. On the other hand this may have boostered study participation and honesty. Given the high level of disclosure we did not feel this relationship negatively influenced participants’ responses. Thirdly, as nearly all study participants had a European ethnicity, further research seems warranted to investigate whether the experiences of this group of donors can be generalized to other ethnic and cultural groups. We acknowledge that the results might not fully represent the experiences of donors from other transplant centres in the Netherlands and beyond. Future studies should ideally be prospective and should include potential donors who withdrew themselves or were not accepted for donation. In addition, it is important to assess whether the transplant professionals perspectives and experiences with regard to unspecified living kidney donation are in line with the donor perspective, in order to create support among transplant professionals to further improve the care for this group of donors.

### Conclusion

In summary, this study showed that our UKDs are generally very satisfied with their donation and, if it were possible, would donate again. Most important complaints about the procedure concerned the length of the donor evaluation and the lack of acknowledgment or resistance for UKDs from both their recipients and health professionals. We call for efforts to optimize the assessment procedures, the education and guidance for UKDs throughout the process, and for more education for transplant professionals about unspecified kidney donation to increase their empathy towards UKDs.

## Data Availability

The raw data supporting the conclusion of this article will be made available by the authors, without undue reservation.
