# Supplementary material for: Twenty Years of Unspecified Kidney Donation: Unspecified Donors Looking Back on Their Donation Experiences
Source: Transpl Int. 2023 Feb 28;36:10959. doi: 10.3389/ti.2023.10959 (PMC10011065; doi:10.3389/ti.2023.10959)
Supplement: Supplementary file 2 [file DataSheet1.docx]

**Interview Guide**

**Pre-donation experiences**

1. How did you experience the pre-donation work-up?
2. Can you tell us about your thoughts and feelings during this period?
3. When you were thinking about donating your kidney and had decided to proceed, how did members of your social network react?
4. How did you feel about these reactions?

**Peri-donation experiences**

1. How did you experience the surgery and hospital stay?
2. Can you tell us about your thoughts and feelings during this period?

**Post-donation experiences**

1. How did you experience the recovery period?
2. Can you tell us about your thoughts and feelings during this period?
3. Can you tell us about the support you received during the donation process?
4. What kind of support did you receive?
5. Was this sufficient for you?
6. If you could go back in time, with the knowledge you have today, would you choose to become an unspecified kidney donor again?
   Also to donate anonymously?

**Experiences with anonymity**

1. How do you feel about the anonymity of your donation?
2. To what extent did the anonymity of the donation influence your donation experience?
3. Did you have contact with the recipient of your kidney in any way? (written, (incidental) meetings, thank you card)
4. How did you experience the contact you had with the recipient?
5. In case of a meeting: Did you have certain expectations with regard to your recipient and to what extent did the recipient meet these expectations?

**Reasons for non-participation (n=20)**

| **Reason (multiple reasons possible)** | **n** |
| --- | --- |
| No interest | 1 |
| Costs too much energy | 1 |
| Lives abroad | 1 |
| Already achieved closure, put the donation behind them | 1 |
| Recent stress | 2 |
| Does not want to participate in research any more | 2 |
| No reason given | 2 |
| No time | 3 |
| Going well with the donor therefore does not feel the need to participate | 4 |
| Medical issues self or partner | 5 |
| Dissatisfaction | 7 |
